# Efficacy of Single and Multiple Injections of Human Umbilical Tissue-Derived Cells following Experimental Stroke in Rats

**DOI:** 10.1371/journal.pone.0054083

**Published:** 2013-01-14

**Authors:** Amjad Shehadah, Jieli Chen, Brian Kramer, Alex Zacharek, Yisheng Cui, Cynthia Roberts, Mei Lu, Michael Chopp

**Affiliations:** 1 Department of Neurology, Henry Ford Hospital, Detroit, Michigan, United States of America; 2 Advanced Technologies and Regenerative Medicine, LLC, Somerville, New Jersey, United States of America; 3 Department of Biostatistics and Research Epidemiology, Henry Ford Hospital, Detroit, Michigan, United States of America; 4 Department of Physics, Oakland University, Rochester, Michigan, United States of America; University of Münster, Germany

## Abstract

**Introduction:**

Human umbilical tissue-derived cells (hUTC) are a promising source of cells for regenerative treatment of stroke. In this study, we tested the efficacy of hUTC in experimental stroke and whether multiple injections of hUTC provide additional therapeutic benefits as compared to a single injection.

**Methods:**

Adult male Wistar rats were subjected to 2 hours of middle cerebral artery occlusion (MCAo), and randomly selected animals were injected (i.v) with 3×10^6^ hUTC or with vehicle control (at day: 1, 1&3 or 1&7 after MCAo, n = 8–9/group). A battery of functional outcome tests was performed at days 1, 7, 14, 21, 28, 35, 42, 49, 56 and 63 after MCAo. Rats were sacrificed at 63 days after MCAo and lesion volumes were measured. To investigate the underlying mechanism of hUTC treatment of stroke, Von Willebrand Factor (vWF), and Synaptophysin immunostaining were performed.

**Results:**

All hUTC treated groups, single or multiple injections, had better functional recovery compared to control (p<0.01). There was no statistically significant difference between a single and multiple injections of hUTC (p = 0.23) or between different multiple injections groups (p>0.07) in functional outcome. All hUTC treatment groups showed significant increases in Synaptophysin, vascular density and perimeter compared to the control group (p<0.05). There was no statistically significant difference between a single and multiple injections of hUTC or between the two groups of multiple injections in all immunohistochemical measurements (p>0.1).

**Conclusion:**

hUTC treatment significantly improves long term functional outcome after stroke and promotes vascular density and synaptic plasticity. At the proscribed doses, multiple injections of hUTC were not superior to single injection therapy in both functional outcome and histological assessments.

## Introduction

Stroke remains the number one cause of disability and the third leading cause of death among Americans every year. Tissue plasminogen activator (tPA), the current treatment for ischemic stroke, while efficacious, is only effective if administered within 4.5 hours of the ischemic event [1], [2]. Therefore, there is a clear and unmet need to develop effective treatments with a wide therapeutic window capable of restoring neural function and reducing disabilities associated with stroke.

Stem cell transplantation to restore neurological function after stroke is a potential therapy [3]–[5]. However, there is a paucity of studies testing whether multiple injections of cells are superior to single injection [6]_._

Cell-based treatments of stroke include neural stem cells, umbilical cord blood cells, bone marrow-derived mesenchymal stem cells (MSCs) [7]–[10], and human umbilical tissue-derived cells (hUTC) are promising sources of cells for regenerative treatment of stroke [11]–[13]. In contrast to other cell types (MSCs, embryonic stem cells etc.), hUTC are obtained by non-invasive methods and their use does not evoke ethical concerns.

In this study, we tested the efficacy of hUTC treatment when administered intravenously 24 hours after experimental stroke in rats and whether multiple injections of hUTC provides any additional beneficial effects as compared to a single injection.

## Materials and Methods

This study was carried out in strict accordance with the recommendations in the Guide for the Care and Use of Laboratory Animals of the National Institutes of Health. The protocol was approved by the Henry Ford Health System’s Institutional Animal Care and Use Committee (IACUC approval number: 1027). All surgery was performed under isoflurane anesthesia, and all efforts were made to minimize suffering.

### Preparation of hUTC

Human umbilical tissue derived cells (hUTC) were provided by Advanced Technologies and Regenerative Medicine, LLC. Cells were isolated and banked as previously described [12]. On the day of treatment, hUTC were thawed in a 37°C water bath and counted using a hemocytometer. Cells were diluted with cell cryopreservation buffer (Janssen R&D) containing 10% DMSO (Sigma, St. Louis) for 3×10^6^/2 ml for injection. Cell viability was measured and exceeded 80%.

hUTC (3×10^6^ cells in 2 ml) or vehicle control (cell cryopreservation buffer, 2 ml) were delivered intravenously at 1 day; 1 and 3 days (1&3d); or 1 and 7 days (1&7d) after stroke.

### Middle Cerebral Artery Occlusion (MCAo) Model and Experimental Groups

Adult male Wistar rats weighing 270–300 g were used in all experiments. Transient right MCAo was induced for 2 hours by advancing a 4–0 surgical nylon suture (18.5–19.5 mm) determined by the animal weight, with its tip rounded by heating near a flame, to block the origin of the middle cerebral artery (MCA), using a method of intra-luminal vascular occlusion modified in our laboratory [14]. Rectal temperature was maintained at 37°C throughout the surgical procedure using a feedback regulated water heating system.

To test the efficacy of single and multiple doses of hUTC, rats were subjected to 2 hrs of transient MCAo and randomly selected animals were injected, via a tail vein, with:

1. 3×10^6^ hUTC in 2 ml administered at 1 day after MCAo (3 M/1d; n = 8).
2. 3×10^6^ hUTC in 2 ml administered at 1 and 3 days after MCAo (3 M/1&3d; n = 8).
3. 3×10^6^ hUTC in 2 ml administered at 1 and 7 days after MCAo (3 M/1&7d; n = 8).
4. Vehicle control (2 ml) administered at 1 day after MCAo (n = 9).
5. Vehicle control (2 ml) administered at 1 and 3 days after MCAo (n = 8).
6. Vehicle control (2 ml) administered at 1 and 7 days after MCAo (n = 9).

All rats were sacrificed at 63 days after MCAo.

### Functional Tests

For each experimental animal, we performed functional tests at one day prior to MCAo and at one day after MCAo immediately prior to the first treatment (baseline), and weekly thereafter (7, 14, 21, 28, 35, 42, 49, 56 and 63 days after MCAo) by an investigator who was blinded to the experimental groups. The functional tests included a modified neurological severity score (mNSS), a foot- fault test and an adhesive removal test.

#### Modified neurological severity score (mNSS)

Modified neurological severity score (mNSS) is a composite of motor, sensory, balance and reflex tests. mNSS is graded on a scale of 0 to 18 (normal score 0; maximal deficit score 18). One score point is awarded for the inability to perform the test or for the lack of a tested reflex; thus, the higher the score, the more severe is the injury [9], [15].

#### Foot-fault test

Foot-fault is a test for placement dysfunction of forelimbs. Animals were placed on an elevated grid floor (45 cm×30 cm), 2.5 cm higher than a solid base floor, with 2.5 cm×2.5 cm diameter openings. Animals tend to move on the grid with their paws placed on the wire frame. When animals inaccurately place a paw, the front limb falls through one of the openings in the grid. When the paw falls or slips between the wires, this was recorded as a foot fault. A total 100 of steps (movement of each forelimb) were counted, and the total number of foot faults for the left forelimb was recorded. The percentages of foot faults are normalized to the number of total counted steps (100 steps) [16], [17].

#### Adhesive-removal somatosensory test

An adhesive removal test was used to measure somatosensory deficits [18], [19]. All rats were familiarized with the testing environment. In brief, two small pieces of adhesive-backed paper dots (of equal size, 113.1 mm^2^) were used as bilateral tactile stimuli occupying the distal-radial region on the wrist of each forelimb. The rats were then returned to their cages. The times to remove each stimulus from both forelimbs were recorded on 3 trials per day. Individual trials were separated by at least 5 min.

### Histological and Immunohistochemical Assessment

At 63 days after MCAo, animals were sacrificed and subsequently brains were fixed by transcardial perfusion with saline containing heparin (2 units/ml), followed by perfusion and immersion in 4% Paraformaldehyde before being embedded in paraffin.

*Lesion volume measurement:* Seven coronal sections of tissue were processed and stained with hematoxylin and eosin (H&E) for calculation of volume of cerebral infarction [20]. The indirect lesion area, in which the intact area of the ipsilateral hemisphere was subtracted from the area of the contralateral hemisphere, was calculated using the Global Lab Image analysis system (Data Translation, Malboro, MA) [20]. The percentages of infarct volume are normalized to the contralateral hemisphere and lesion volume is presented as a volume percentage of the lesion compared with the contralateral hemisphere.

### Immunohistochemical Staining

A standard paraffin block was obtained from the center of the lesion (bregma –1 mm to +1 mm). A series of 6 µm thick sections were cut from the block. Brain sections obtained from hUTC treatment and vehicle control groups were used for immunostaining. Immunostaining was performed using antibodies against Von Willebrand Factor (vWF), an endothelial cell marker (1∶400; Dako, Carpenteria, CA) and Synaptophysin (1∶500, Chemicon).

#### Quantitation

For measurement of vascular density and perimeters, vWF immunostained coronal sections were used. Eight fields of view from the ischemic boundary zone (IBZ) were digitized using a 40X objective (Olympus BX40) via the MCID computer imaging analysis system (Imaging Research, St. Catharines, Canada). The number of vessels and their perimeters were counted throughout each field of view. The total number of vessels was divided by the total tissue-area to determine vascular density. Data are presented as total number of vessels per mm^2^.

For semi-quantification of Synaptophysin, immunostained coronal sections were used. Eight fields of view from the IBZ in each section were digitized under a 40x objective. The positive area was measured. Data are presented as percentage of positive area.

All cell counts were performed by observers blinded to the individual treatment status of the animals.

### Statistical Analysis

Rats subjected to MCAo were randomized into one of the six groups. Animals were treated with hUTC (3×10^6^ cells in 2 ml) or vehicle control (2 ml) intravenously at 1 day; 1 and 3 days (1&3d); or 1 and 7 days (1&7d) after stroke.

Behavior tests (adhesive test, foot-fault test and mNSS) were performed prior to MCAo, day 1 after MCAo prior to the study treatment (baseline), and at days 7, 14, 21, 28, 35, 42, 49, 56, and day 63 after MCAo. Histology evaluation was processed after sacrifice at day 63.

This study investigated the efficacy of hUTC treatment on the functional recovery (primary outcome), measured from three behavioral tests, and the histological evaluation (secondary outcomes). Data were evaluated for normality. Data transformation was considered if data were not normal. If the data were not distributed normally, the ranked data were used for the analysis. We first tested for differences between the three controls groups (group 4 to 6), if none were found; the controls would be combined as co-control to increase statistical power.

The global test using Generalize Estimating Equation (GEE) [21] was implemented to test the group difference on functional recovery measured from the three behavioral tests (adhesive test, foot-fault test and mNSS). The global test on multiple outcomes is more efficient than a single outcome, when the group effects are consistent on all the outcomes (e.g., the positive correlation). The analysis of the treatment effect started by testing for overall global effect and followed by a subgroup comparison for each individual behavioral test, if the overall global effect was detected at the 0.05 level. Otherwise, subgroup analysis would be considered as exploratory. Individual functional outcome within different groups and days was tested using contract statement.

One-way ANOVA was used to test the group difference on histology measurements at day 63. Correlation coefficients were calculated among the histology measurements as well as the correlation to the functional outcome at day 63 adjusting for treatment groups.

## Results

### Neurological Functional Outcome and Lesion Volume (Figure 1)

#### Functional outcome

To test whether hUTC treatment of stroke rats regulates functional outcome, a battery of functional tests was performed. Functional response was measured for each experimental animal, at day 1 (before treatment) and at 7, 14, 21, 28, 35, 42, 49, 56 and 63 days after MCAo.

All rats exhibited the same level of neurological functional deficits post stroke at 24 h immediately prior treatment with no significant differences among the groups (p>0.09).

Since the three control groups did not show significant differences in functional outcome from day 1 to day 63 after MCAo, the three control groups were combined into one as co-control (n = 26).

A significant overall group test based on the global test (p<0.0001) indicated that effects on functional recovery were different among the 4 groups from day 14 to 63. All hUTC treatments, single (1d) or multiple (1&3d and 1&7d), significantly improved functional recovery compared to co-control (p<0.01).

Rats receiving hUTC at 1d and at 1&7d showed significant improvement in neurological function from 14 days to 63 days compared with co-control group (p<0.05). hUTC treatment at 1&3d significantly improved functional outcome from day 7 to 63 compared to the co-control group (p<0.05).

**Figure 1:**
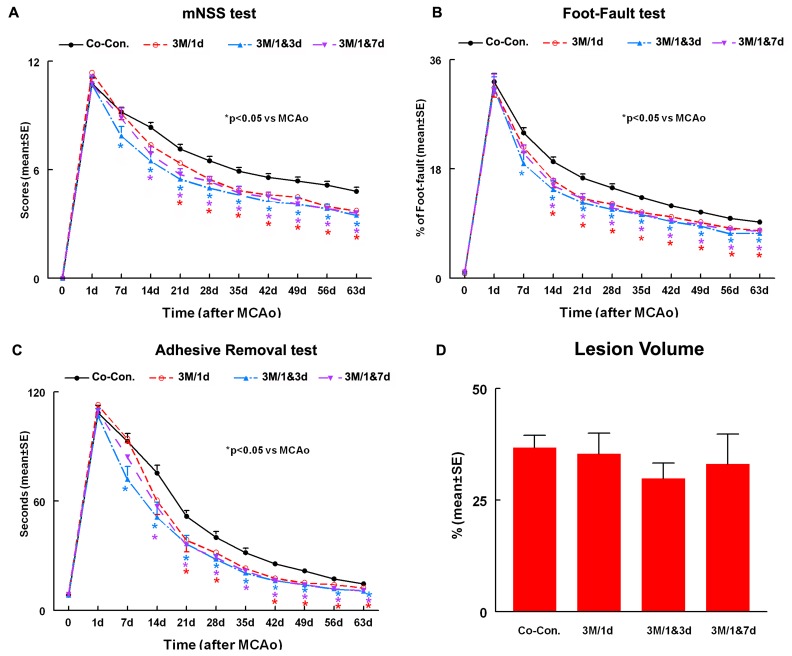
Neurological outcome and lesion volume measurements after stroke. Panels **A–C** show mNSS (**A**) foot-fault (**B**) and adhesive-removal (**C**) tests after stroke in the 4 experimental groups: 1. Combined Control (Co-Con.). 2. 3×10^6^ hUTC administered at 1 day after MCAo (3M/1d). 3. 3×10^6^ hUTC administered at 1 and 3 days after MCAo (3M/1&3d). 4. 3×10^6^ hUTC administered at 1 and 7 days after MCAo (3M/1&7d). Panel **D** shows the lesion volume in the 4 experimental groups. SE = standard error. *p<0.05 vs Co-control.

The data for subgroup analysis for each individual functional test (foot fault, mNSS and adhesive removal tests) within different groups and days is presented in Figure 1.

There was no statistically significant difference between single and multiple injections of hUTC (p = 0.23) or between the two groups of multiple injections (1&3d vs. 1&7d, p>0.07) at all testing time points. Treatment with multiple injections of hUTC at 1&3d marginally improved functional outcome at days 56 (p = 0.08) and 63 (p = 0.07) compared to a single injection of hUTC given at day 1.

#### Lesion volume

No significant differences of ischemic lesion volumes in the hUTC treatment groups were detected compared with the co-control group (**Figure 1D**, p>0.05).

### Synaptophysin Expression in the IBZ

Synaptophysin is a marker for presynaptic plasticity and synaptogenesis [22]. To test whether hUTC cell treatment regulates synaptic plasticity, Synaptophysin immunostaining was performed and the immunoreactive positive area was measured in the ischemic border.

**Figure 2:**
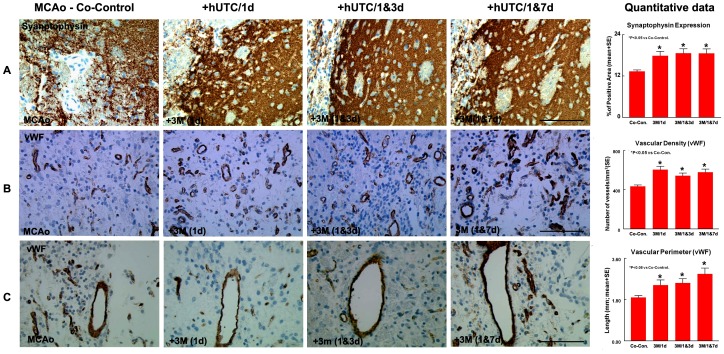
hUTC treatment effect on vascular density, perimeter and synaptophysin expression. Panel **A** shows significant increase in Synaptophysin expression in the IBZ in all hUTC treatment groups compared to the co-control group. Panels **B–C** show significant increase in vascular density and perimeter in the IBZ in all hUTC treatment groups compared to the co-control group. *p<0.05 vs Co-control. SE = standard error. Scale bar in A,B and C = 100µm.

All hUTC treatment groups showed significant increases in Synaptophysin expression in the IBZ compared to the co-control group (p<0.05, **Figure 2A**). Synaptophysin expression in the IBZ was marginally correlated with the adhesive removal test (r = −0.26, p = 0.053). There was no statistically significant difference between single and multiple injections of hUTC or between the two groups of multiple injections in Synaptophysin expression in the IBZ (p>0.6).

### Vascular Density and Perimeter in the IBZ

To measure vascular perimeter and density, we performed vWF immunostaining. All hUTC treatment groups showed a significantly higher vascular density when compared to co-control group (p<0.05, **Figure 2B**). There was no statistically significant difference between single and multiple injections of hUTC or between the two groups of multiple injections in vascular density in the IBZ (p>0.1).

All hUTC treatment groups showed significantly increased vascular perimeters compared to the co-control group (p<0.05, **Figure 2C**). There was no statistically significant difference in vascular perimeter in the IBZ between the two groups of multiple injections (p = 0.13). However, there was marginal but not significant difference in perimeter between 3M/1d and 3M/1&7d (p = 0.07).

## Discussion

Cell therapy has shown promising therapeutic potential in myocardial [23], limb [24], and brain ischemia [25]. Bone marrow-derived MSCs have been extensively studied in animal models of stroke and shown to promote neurovascular remodeling concomitantly with a significant improvement in neurological outcome [15], [26], [27]. However, little has been done to address the clinically relevant question whether treatment of stroke with multiple injections of cells are superior to a single injection. This is the first study to address the question whether multiple injections of hUTC when initiated 24 hours after experimental stroke in rats provides any additional beneficial effects as compared to a single injection.

Determining the optimal therapeutic protocol using stem cells for stroke therapy would enable us to improve the design of clinical trials to test the efficacy of hUTCs treatment in stroke patients. It is clearly beneficial to know whether multiple injections are superior to a single injection, as a single injection is more convenient, faster, requires lower number of cells and most likely, is accompanied with lower risks.

Omori et al. [6] compared the effects of a single intravenous injection of human MSCs (1×10^6^ or 3×10^6^) at a single time point (6 hours) with a low dose of 1×10^6^ human MSCs injected at multiple time points (6 h, 24 h and 48 h or 6 h, 24 h and 1 week) after MCAo. Their results showed that the greatest therapeutic benefit was achieved following a single 3×10^6^ cell dose injection at 6 hours post MCAo, rather than multiple lower cell infusions over multiple time points [6]. In a different study, injection of bone marrow cells at day 3 post experimental myocardial infarction in mice reduced infarct size and improved left ventricular function, while multiple injections (at day 3 and 7 or 3, 7 and 14) post myocardial infarction had no additive effect [28].

In a dose escalation study, Zhang et al. [13] demonstrated that endogenous neurorestorative response and neurological recovery were augmented after a single intravenous administration of hUTC at dose 3×10^6^. Statistically significant improvements after intravenous administration of hUTC treatment were observed when treatment was initiated up to 30 days but not when hUTC were administered at 90 days after stroke [13]. Our data demonstrate that treatment of stroke with 3×10^6^ hUTC improves functional outcome compared to control when treatment is initiated at 24 hours after MCAo. However, there was no statistically significant difference between single and multiple injections of hUTC or between the two groups of multiple injections both in functional outcome and immunohistochemical measurements. This may be due to ceiling effect with 3×10^6^ hUTC providing the optimal number of cells to induce neurorestorative effects with no added benefit observed by multiple injections. Our data thus suggest that treatment with multiple injections is not superior to a single injection.

hUTC treatment was associated with a significantly higher vascular density and increased expression of the synaptic protein, Synaptophysin, when compared to the vehicle control group. Treatment of stroke with hUTC improved functional outcome with no change in the volume of the cerebral infarction. Thus, functional outcome after hUTC treatment likely results from a neurorestorative effect rather than neuroprotective effect when hUTC is administered 24 h after MCAo.

### Vascular Density and Perimeter after hUTC Treatment

The adult brain vascular system is activated in response to pathological conditions including stroke [29]. Cerebral blood flow regulation is a critical step in maintaining neural function [30]. The formation of new blood vessels via angiogenesis is important in the pathophysiology of vascular disease [31]. In the rodent brain, capillary sprouting is initiated at the border of the infarct and new vessels develop in the ischemic boundary zone [32], [33].

Stroke patients with a greater cerebral blood flow appear to have a better outcome than patients with lower flow [34]–[36]. Neurorestorative treatments, either cell-based or pharmacological therapies, in animal models of stroke increase angiogenesis which is associated with and may underlie improvements in functional outcome [37].

Our data demonstrate that hUTC treatment improves functional recovery and has a positive influence on the vascular density and perimeter in the IBZ. Treatment with hUTC may enhance recovery after stroke through modulation of the brain vascular system.

### Synaptophysin Expression after hUTC Treatment

Synaptic plasticity is an important mediator of functional recovery after stroke [38], [39]. Functional plasticity in the motor cortex is accompanied by changes in dendritic and synaptic structure, as well as by alterations in the regulation of cortical neurotransmitter systems [39], [40].

Synaptophysin is a presynaptic vesicle protein that is found in all nerve terminals [41].

Synaptophysin is used to quantify numbers of terminals during neuroanatomical remodeling and neural development [42]–[45].

Neurorestorative treatments of stroke increase synaptic plasticity in the IBZ [46], [47] as evidenced by the increased expression of synaptic proteins such as Synaptophysin and growth-associated protein 43 [41]. Reduction of neurological deficits after stroke has been attributed to extensive synaptic and functional reorganization.

Stroke rats treated with hUTC demonstrated significant increase in Synaptophysin expression in the ischemic brain compared to co-control. Functional benefits derived from hUTC treatment of stroke suggest an effect of hUTC on synaptic plasticity.

In summary, the data indicate that hUTC treatment started 24 h after MCAo significantly improves long term functional outcome after stroke and increases vascular density, perimeter and synaptic plasticity in the ischemic brain. Multiple injections of hUTC were not superior to single injection therapy in both functional outcome and histological assessments.
